# Tissue Specific Reference Genes for MicroRNA Expression Analysis in a Mouse Model of Peripheral Nerve Injury

**DOI:** 10.3389/fnmol.2019.00283

**Published:** 2019-11-22

**Authors:** Theodora Kalpachidou, Kai K. Kummer, Miodrag Mitrić, Michaela Kress

**Affiliations:** Institute of Physiology, Department of Physiology and Medical Physics, Medical University of Innsbruck, Innsbruck, Austria

**Keywords:** miRNA normalization, ncRNA, spared nerve injury, dorsal root ganglia, spinal dorsal horn, medial prefrontal cortex

## Abstract

MicroRNAs (miRNAs) have emerged as master switch regulators in many biological processes in health and disease, including neuropathy. miRNAs are commonly quantified by reverse transcription quantitative real-time polymerase chain reaction (RT-qPCR), usually estimated as relative expression through reference genes normalization. Different non-coding RNAs (ncRNAs) are used for miRNA normalization; however, there is no study identifying the optimal reference genes in animal models for peripheral nerve injury. We evaluated the stability of eleven ncRNAs, commonly used for miRNA normalization, in dorsal root ganglia (DRG), dorsal horn of the spinal cord (dhSC), and medial prefrontal cortex (mPFC) in the mouse spared nerve injury (SNI) model. After RT-qPCR, the stability of each ncRNA was determined by using four different methods: BestKeeper, the comparative delta-Cq method, geNorm, and NormFinder. The candidates were rated according to their performance in each method and an overall ranking list was compiled. The most stable ncRNAs were: sno420, sno429, and sno202 in DRG; sno429, sno202, and U6 in dhSC; sno202, sno420, and sno142 in mPFC. We provide the first reference genes’ evaluation for miRNA normalization in different neuronal tissues in an animal model of peripheral nerve injury. Our results underline the need for careful selection of reference genes for miRNA normalization in different tissues and experimental conditions. We further anticipate that our findings can be used in a broad range of nerve injury related studies, to ensure validity and promote reproducibility in miRNA quantification.

## Introduction

MicroRNAs (miRNAs) are small non-coding RNAs (ncRNAs) of approximately 22 nucleotides length, which can regulate gene expression by translational inhibition or promotion of degradation of their target mRNAs (Eulalio et al., 2008). miRNAs play fundamental roles in biological processes, such as cell proliferation, differentiation, and survival and are crucial for normal developmental processes, homeostasis, as well as a plethora of diseases and pathologies (Vidigal and Ventura, 2015). Accumulating data suggest that miRNA expression patterns are deregulated following peripheral nerve injury (Kress et al., 2013; Sakai and Suzuki, 2015; Jiangpan et al., 2016; Lopez-Gonzalez et al., 2017). Furthermore, several studies have identified miRNAs that are upregulated upon peripheral nerve injury and can promote regeneration (Strickland et al., 2011; Zhou et al., 2012; Wu and Murashov, 2013; Motti et al., 2017), rendering miRNAs attractive potential targets for therapeutic interventions (Jiangpan et al., 2016; Ghibaudi et al., 2017; Zhang J. et al., 2018). To this end, in depth mechanistic understanding of the role of up- and downregulated miRNAs in human pathologies and mouse models of the respective disorders is increasing, which in turn necessitates the reliable and reproducible quantification of miRNA expression levels.

miRNA expression is commonly assessed by reverse transcription quantitative real-time polymerase chain reaction (RT-qPCR), either as a primary method or for the validation of miRNA profiling results (e.g., miRNA microarrays, small RNA sequencing). RT-qPCR highly relies on appropriate normalization procedures, usually achieved by means of relative expression to one or multiple reference genes (Bustin et al., 2009). In order to qualify as reference genes, the expression of such genes needs to remain stable when exposed to specific experimental conditions (Kozera and Rapacz, 2013). It is commonly accepted that no gene exists for which expression levels remain unaltered under every experimental condition and in every tissue or cell line. The importance of using appropriate reference genes is stressed in the Minimum Information for publication of Quantitative real-time PCR Experiments (MIQE) guidelines, which state that “normalization against a single reference gene is not acceptable unless the investigators present clear evidence for the reviewers that confirms its invariant expression under the experimental conditions described. The optimal number and choice of reference genes must be experimentally determined and the method reported” (Bustin et al., 2009).

Based on these considerations, several publications have analyzed the stability of potential reference genes for miRNA expression normalization in different human, animal, and plant studies (Bouhaddioui et al., 2014; Matouskova et al., 2014; Ferdous et al., 2015; Wu et al., 2016; Babion et al., 2017; Mase et al., 2017). To our knowledge, the present study for the first time evaluated potential reference genes in the mouse spared nerve injury (SNI) model, a commonly used, robust and reproducible animal model of peripheral nerve injury (Decosterd and Woolf, 2000). We analyzed the expression of 11 ncRNAs, which are frequently used as reference genes, with commercially available assays in the dorsal root ganglia (DRG) and dorsal horn of the spinal cord (dhSC) ipsilateral to the injury as well as the medial prefrontal cortex (mPFC) contralateral to the injury, using four different statistical methods: BestKeeper (Pfaffl et al., 2004), the comparative delta-Cq method (Silver et al., 2006), geNorm (Vandesompele et al., 2002), and NormFinder (Andersen et al., 2004). Upon rating each ncRNA according to its performance in the different methods, we compiled an overall ranking list and propose the most appropriate reference genes for assessing miRNA expression in the SNI mouse model in three neuronal tissues.

## Materials and Methods

### Animals

In total, 16 male, 8–12 weeks old C57Bl/6J mice (Janvier Labs, France) were used for the experiments. Animals were kept under standard pathogen free (SPF) conditions, at 24°C on a 12 h light/dark cycle and had free access to autoclaved pelleted food and water. All mice were treated in accordance with the Ethics Guidelines of Animal Care (Medical University of Innsbruck), the European Communities Council Directive of 22nd September 2010 on the protection of animals used for scientific purposes (2010/63/EU) and all procedures were approved by the Austrian National Animal Experiment Ethics Committee of the Austrian Bundesministerium für Wissenschaft und Forschung (permit number BMWF-66.011/0054-WF/V/3b/2015).

### Spared Nerve Injury Model

Mice were divided in three groups, non-treated (*n* = 5), sham (*n* = 6), and SNI (*n* = 5). The SNI model was adopted from Decosterd and Woolf (2000). Briefly, mice were anesthetized with a mixture of xylazine (10 mg/kg, AniMedica, Germany) and ketamine (100 mg/kg, Graeub, Switzerland). The skin of the lateral surface of the left thigh was incised and the sciatic nerve was exposed. For the SNI procedure, the common peroneal and the tibial nerves were ligated with 4-0 vicryl (Sh-1 plus, Ethicon, Austria) and a portion of approximately 1–2 mm length was excised, leaving the sural nerve intact. Mice subjected to sham surgery had their sciatic nerve exposed but not lesioned. After surgery, the skin was sutured using 4-0 vicryl and mice were placed on a heat block adjusted to 37°C until recovery. Non-treated mice were not subjected to any treatment.

### Tissue Collection

Non-treated, sham and SNI mice 7 days post-surgery (only for sham and SNI mice) were deeply anesthetized with isoflurane (Forane, Abbott, United Kingdom) and euthanized by decapitation. Lumbar ipsilateral L3-L5 DRG and dhSC as well as contralateral mPFC were microdissected, snap-frozen in liquid nitrogen, and stored at –80°C until use.

### RNA Extraction

RNA extraction was performed using peqGOLD TriFast reagent (Peqlab Biotechnologie, Germany), according to the protocol provided by the manufacturer [chloroform (C2432) and absolute ethanol (107017) were obtained from Merck, United States]. The air-dried RNA pellets were diluted in nuclease free water (R0582, ThermoFisher Scientific, United States). RNA quantity was estimated using Nanodrop 2000 (ThermoFisher Scientific) and RNA integrity [RNA integrity number (RIN)] was assessed using 2100 Bioanalyzer (Agilent Technologies, United States) in the deep-sequencing core facility of the Medical University of Innsbruck.

### Reverse Transcription and Quantitative Real-Time PCR

We evaluated the stability of all ncRNAs, suggested as controls and available by Thermo Fisher Scientific. The expression of small nucleolar (snoRNA) and small nuclear (snRNA) RNAs, as potential reference genes, was quantified using Taqman MicroRNA Control Assays, which through a two-step protocol ensure high specificity and sensitivity and do not require prior DNase treatment of the RNA template, since the stem-loop transcription amplifies only the mature sequence (Mestdagh et al., 2008). Reverse transcription and qPCR reactions were prepared according to the protocol provided by the supplier. Briefly, each reverse transcription reaction contained 10 ng of total RNA, 1X reverse transcription buffer, 5.5 mM MgCl_2_ (GeneAmp^®^ 10X PCR Buffer II and MgCl_2_, #N8080130), 1 mM dNTPs, RNase inhibitor (#N8080119), 50 units of MultiScribe^TM^ Reverse Transcriptase (#4311235), and 1X RT specific primers (Table 1), in a final volume of 15 μL, adjusted with nuclease free water (#R0582). The RT program was: 30 min at 16°C, 30 min at 42°C, 5 min at 85°C, followed by a holding step at 4°C. After reverse transcription, reactions were stored at –20°C until next day. Each qPCR reaction contained 1.33 μL of the RT product, 1X TaqMan^®^ Universal Master Mix II, no UNG (#44440049), 1X of the appropriate assay (Table 1), and nuclease free water up to a final volume of 20 μL. Reactions for each sample were prepared as technical duplicates, alongside reverse transcription non-template controls, loaded on MicroAmp Fast Optical 96-well reaction plates (#4346906) and placed in the 7500 Fast RT-PCR system (all reagents for RT-qPCR were from ThermoFisher Scientific). The PCR cycle protocol was: 10 min at 95°C, 40 two-step cycles of 15 s at 95°C, and 1 min at 60°C. In order to account for potential methodological variabilities, all RT (day 1) and qPCR reactions (day 2) per tissue assessed were processed on the same day. The MIQE checklist for authors, reviewers, and editors can be found in Supplementary Table S1.

**TABLE 1:** Details of the ncRNA assays used.

**Assay name**	**Assay ID**	**Official symbol**	**Official name**	**Also known as**	**RNA class**
snoRNA55	001228	Snord110	small nucleolar RNA, C/D box 110	MBII-55	Small nucleolar RNA
snoRNA135	001230	Snord65	small nucleolar RNA, C/D box 65	MBII-135; snoRNA135	Small nucleolar RNA
snoRNA142	001231	Snord66	small nucleolar RNA, C/D box 66	MBII-142; snoRNA142	Small nucleolar RNA
snoRNA202	001232	Snord68	small nucleolar RNA, C/D box 68	MBII-202	Small nucleolar RNA
snoRNA234	001234	Snord70	small nucleolar RNA, C/D box 70	MBII-234; snoRNA234	Small nucleolar RNA
snoRNA251	001236	Snord85	small nucleolar RNA, C/D box 85	Z50; MBII-251	Small nucleolar RNA
snoRNA292	001242	Snord42a	small nucleolar RNA, C/D box 42A	U42A; MBII-292	Small nucleolar RNA
snoRNA412	001243	Snord45b	small nucleolar RNA, C/D box 45B	MBII-412	Small nucleolar RNA
snoRNA420	001239	Snord99	small nucleolar RNA, C/D box 99	MBII-420	Small nucleolar RNA
snoRNA429	001240	Snord100	small nucleolar RNA, C/D box 100	Z51; MBII-429	Small nucleolar RNA
U6 snRNA	001973	Rnu6	U6 small nuclear RNA	−	Small nuclear RNA
hsa-miR-21	000397	Mir21a	microRNA 21a miRBase: MIMAT0000530	mmu-miR21a-5p	Small non-coding RNA

### Data Analysis

Results were extracted from the 7500 Software v2.3 (ThermoFisher Scientific), by manually setting the threshold at 0.1 (single threshold method) for all amplicons and keeping the automatic baseline (baseline start cycle: 3, baseline end cycle: 15). Additionally, raw amplification data were imported to LinRegPCR program (Ramakers et al., 2003; Ruijter et al., 2009, 2015; Tuomi et al., 2010), which performs a baseline correction of the amplification data, determines a window of linearity, and through a linear regression analysis determines the qPCR efficiency and the Cq value per reaction, in order to estimate the mean PCR efficiency per primer set or assay [52]. The efficiencies for each amplicon were calculated using LinRegPCR (v2014) for hydrolysis probes with the following parameters: window of linearity: four points; exclude no plateau samples; include efficiency outlier samples; log-linear phase criterion: strictly continuous log-linear phase. Mean Cq values for each sample and ncRNA were calculated as the average of the technical duplicates. The stability of all evaluated ncRNAs was estimated based on the Cq values obtained from single threshold settings and LinRegPCR program adjustments, by using four different statistical approaches that are commonly used for stability assessment of reference genes: BestKeeper (Pfaffl et al., 2004), the comparative delta-Cq method (Silver et al., 2006), geNorm (Vandesompele et al., 2002), and NormFinder (Andersen et al., 2004).

For BestKeeper (Pfaffl et al., 2004), the appropriate reference genes need to exhibit low variability and comparable levels of expression. Variability was defined by the standard deviation (SD) of mean Cq values and low SD values corresponded to stable ncRNAs. Candidates with SD > 1 were considered inappropriate for normalization. Similar levels of expression were explored using Pearson’s linear correlation, by comparing each ncRNA with the BestKeeper index (BKI), which was the geometric mean of the mean Cq values of all evaluated ncRNAs. The closer the correlation was to 1, the more stably an ncRNA was considered to be expressed. A mean ranking score for each ncRNA was calculated by averaging the scores obtained by SD and correlation with the BKI rankings.

In the comparative delta-Cq method (Silver et al., 2006), all pairs of ncRNAs were compared with each other, according to their Cq differences (ΔCq). Variability was determined by calculating the average SD between the pairwise comparisons of all ncRNAs and a low average SD indicated increased stability.

GeNorm (Vandesompele et al., 2002) calculated the average expression stability M, which represents the average pairwise variation of each evaluated ncRNA compared to all the rest. Low M values suggested that the ncRNAs expression was stable. Subsequently, geNorm performed repeated stepwise exclusions (by repeatedly omitting the candidate with the worst M value) and identified the most stable pair of ncRNAs. Additionally, geNorm identified the optimal number of reference genes, by calculating the pairwise variation coefficient (V value).

NormFinder (Andersen et al., 2004), besides calculating the variation of expression, estimated the variation between groups. Specifically, for each ncRNA the stability value ρ was estimated as well as the intra- and inter-group variations. The lowest ρ values represented more stably expressed candidates. Furthermore, a stably expressed ncRNA needed to exhibit intra- and inter-group variations as close as possible to zero.

Each ncRNA was subsequently ranked according to its performance in each of the four different methods (with 1 indicating the most stable ncRNA). The overall stability of each candidate was estimated by calculating the mean of the rankings provided by each statistical approach.

The expression levels of miR-21a-5p were quantified in the DRG of non-treated, sham, and SNI mice and expressed as fold changes relative to the respective expression in the non-treated mice using the 2^–ΔΔCq^ method. ncRNAs were assessed for their suitability for the quantification of miR-21a-5p in DRG by using as a reference gene(s): (1) three (sno420, sno429, and sno202) and two (sno420 and sno429) most stable ncRNAs from the overall ranking, (2) two most stable pair of ncRNAs as suggested by geNorm (sno202 and sno420) and Normfinder (sno234 and sno429), and (3) each ncRNA as a single normalizer.

For statistical data analyses, GraphPad Prism 7.00 and IBM SPSS Statistics 21 were used. Statistical tests used were specified in the text or in their respective figures or tables. Statistical analysis for miR-21a-5p expression was performed using one-way analysis of variance (ANOVA) with the group of animals (non-treated, sham, and SNI) as the independent factor, followed by Tukey’s multiple comparison test, when appropriate, in order to identify differences between groups. The level of statistical significance was predefined at *p* < 0.05. Graphs were plotted in GraphPad Prism 7.00 and illustrated in CorelDRAW X7.

## Results

### RNA Quantity and Integrity

RNA quantity and integrity varied in between samples and tissues (Supplementary Table S2). For DRG the median total RNA concentration was 102.3 ng/μL with an interquartile range (IQR) of 88.85–122.6 ng/μL, for dhSC the median RNA concentration was 127.8 ng/μL with an IQR of: 106.2–176.2 ng/μL, and for mPFC the median RNA concentration was 68.35 ng/μL with an IQR of: 58.43–88.95 ng/μL. Analysis of integrity provided median values of RIN = 6.85 (IQR: 6.45–7.55) in DRG, RIN = 7.55 (IQR: 6.825–8.0) in dhSC, and RIN = 7.35 (IQR: 7.1–7.75) in mPFC.

### qPCR Efficiencies

Mean qPCR efficiencies for all evaluated ncRNAs were calculated by the LinRegPCR program (Supplementary Table S3). *R*^2^ values for all amplicons, as calculated by LinRegPCR, were > 0.998. Correlation coefficients for RIN values and amplicons’ efficiencies were not statistically significant (Supplementary Table S4). Furthermore, no statistically significant difference was observed for individual assay performance on different experimental days (Supplementary Table S5A) or in different experimental groups (Supplementary Table S5B), suggesting that all assays performed similarly in all experimental groups, experimental days, and tissues.

### Cq Values Distribution

**FIGURE 1:**
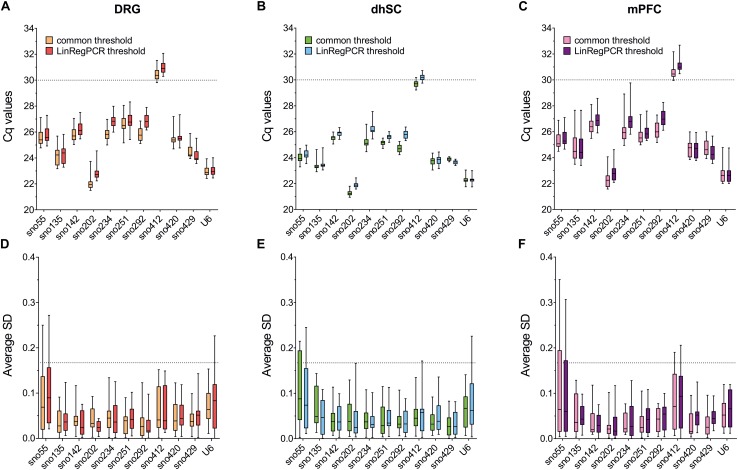
Raw Cq values and average standard deviations of technical duplicates for all evaluated ncRNAs in DRG **(A,D)**, dhSC **(B,E)**, and mPFC **(C,F)** of non-treated (*n* = 5), sham (*n* = 6), and SNI (*n* = 5) mice, as estimated by setting a common threshold (0.1) and by using LinRegPCR software. Values are presented as boxplots, representing upper quartile, median, and lower quartile, whereas whiskers depict minimum and maximum values. The dotted line in **A–C** is set at Cq = 30 and in **D–F** at 0.167. DRG: dorsal root ganglia, dhSC: dorsal horn of the spinal cord, mPFC: medial prefrontal cortex, Cq: quantitation cycle, SD: standard deviation.

After setting the common threshold at 0.1 and after importing the raw amplification data into LinRegPCR, we assessed the raw Cq values (Figures 1A–C). All ncRNAs were successfully amplified and both settings provided similar results in DRG, dhSC, and mPFC. In all three tissues, the most abundantly expressed ncRNA was sno202, followed by U6, whereas the least abundant ncRNA was sno412 (Figures 1A–C and Supplementary Table S6). We further analyzed the SDs of the technical duplicates per sample and amplicon, since reproducibility is critically important for RT-qPCR quantification (Figures 1D–F and Supplementary Table S6). SDs for technical replicates ≤0.167 are required to detect a 2-fold change of expression in 99.7% of cases (Applied Biosystems, 2016). Since sno55 exhibited SDs > 0.167 in the technical duplicates in all three tissues, it was excluded from further analysis. Likewise, sno412 was excluded from assessment in the mPFC. In DRG and dhSC, U6 also showed SDs > 0.167 according to LinRegPCR settings, however, was kept for further analysis, since the common threshold setting suggested appropriate variability in technical replicates.

### Bestkeeper

**FIGURE 2:**
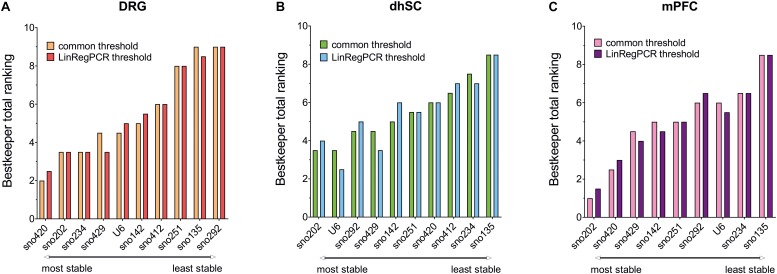
Total ranking obtained from Bestkeeper for each evaluated ncRNA **(A)**, dhSC **(B)**, and mPFC **(C)** of non-treated (*n* = 5), sham (*n* = 6), and SNI (*n* = 5) mice, as estimated by setting a common threshold (0.1) and by using LinRegPCR software. Total ranking was calculated by averaging the rankings achieved by each ncRNA in SD and BKI correlation coefficient. DRG: dorsal root ganglia, dhSC: dorsal horn of the spinal cord, mPFC: medial prefrontal cortex.

Data extracted using both the common threshold and LinRegPCR adjustments, provided similar results in BestKeeper evaluation (Figure 2 and Supplementary Table S7). In DRG (Figure 2A and Supplementary Table S7A), U6 showed the lowest SD (SD = 0.336 for the common threshold and 0.341 for LinRegPCR) across non-treated, sham, and SNI mice, whereas sno251 showed the highest SD (SD = 0.602 for the common threshold and 0.604 for LinRegPCR). All SDs were below the proposed cut-off value of < 1 for the exclusion of a candidate. Correlation analysis between each ncRNA and BKI revealed *r* > 0.92 for sno202, sno420, and sno429, whereas sno135, sno292, and U6 had *r* values <0.67. In the dhSC (Figure 2B and Supplementary Table S7B), sno429 exhibited the lowest SD (0.138 and 0.113, according to the common threshold and LinRegPCR, respectively) and sno234 the highest SD (0.333 using the common threshold and 0.325 according to LinRegPCR). *r* values for most ncRNAs were surprisingly low and *p* values were not significant for sno135, sno142, sno251, sno412, and sno429, indicating no correlation with the BKI. Out of the ncRNAs, which significantly correlated with the BKI, the best correlation was determined for sno292 (*r* = 0.738), followed by U6 (*r* = 0.732) for the common threshold, whereas for LinRegPCR processed data, the best correlation was observed for U6 (*r* = 0.777), followed by sno292 (*r* = 0.735). In the mPFC (Figure 2C and Supplementary Table S7C), sno202 showed the lowest SD (0.494 for the common threshold and 0.498 for LinRegPCR) and sno135 the highest SD (0.900 for the common threshold and 0.905 for LinRegPCR). Furthermore, in the data normalized with the common threshold, sno202 exhibited the highest correlation (*r* = 0.918) with the BKI, whereas in the LinRegPCR processed data, the best correlation was observed for sno429 (*r* = 0.923).

### Comparative Delta-Cq Method

**FIGURE 3:**
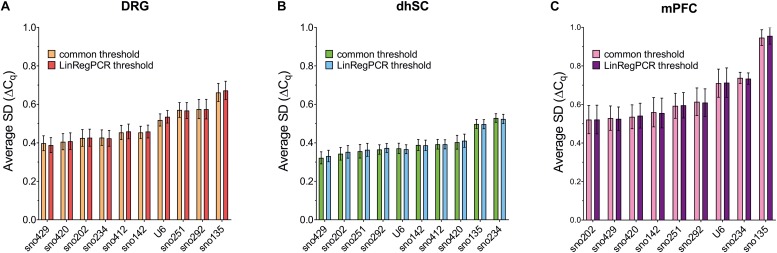
Average standard deviation (SD) of the Cq differences (ΔCq) computed for each evaluated ncRNA vs all the remaining ncRNAs (comparative delta-Cq method) in DRG **(A)**, dhSC **(B)**, and mPFC **(C)** of non-treated (*n* = 5), sham (*n* = 6), and SNI (*n* = 5) mice, as estimated by setting a common threshold (0.1) and by using LinRegPCR software. DRG: dorsal root ganglia, dhSC: dorsal horn of the spinal cord, mPFC: medial prefrontal cortex, SD: standard deviation.

The comparative delta-Cq method provided similar results for both common threshold and LinRegPCR processed data (see Figure 3 and Supplementary Table S8 for details). In DRG, the most stable ncRNA was sno429 (with a ΔCq SD = 0.398 in the common threshold and 0.388 in LinRegPCR), whereas sno135 was the least stable, with ΔCq SD > 0.66 (Figure 3A and Supplementary Table S8A). In dhSC, sno429 showed the lowest ΔCq SD (0.322 in the common threshold and 0.332 in LinRegPCR) and sno234 the highest (0.529 in the common threshold and 0.524 in LinRegPCR; Figure 3B and Supplementary Table S8B). Overall, in the mPFC all ncRNAs had higher ΔCq SDs (Figure 3C and Supplementary Table S8C). Among them, sno202 displayed the highest stability (with a ΔCq SD = 0.521 in the common threshold and equal to 0.522 in LinRegPCR processed data) and sno135 the lowest (ΔCq SD = 0.947 in the common threshold and 0.956 in LinRegPCR data).

### GeNorm

**FIGURE 4:**
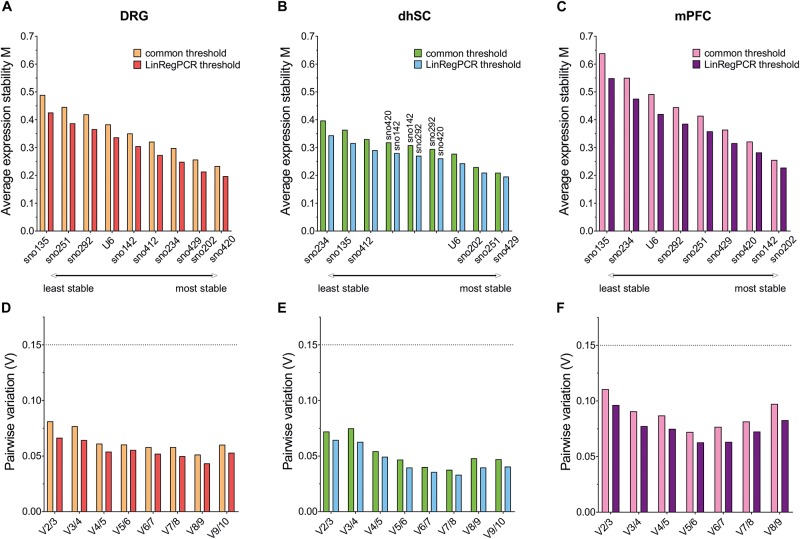
Average expression stability of the remaining ncRNAs after stepwise exclusion of the least stable candidate (upper panel) and pairwise variation analysis (lower panel), as calculated by geNorm for all ncRNAs in DRG **(A,D)**, dhSC **(B,E)**, and mPFC **(C,F)** of non-treated (*n* = 5), sham (*n* = 6), and SNI (*n* = 5) mice, as estimated by setting a common threshold (0.1) and by using LinRegPCR software. The dotted line represents a pairwise variability of 0.15. DRG: dorsal root ganglia, dhSC: dorsal horn of the spinal cord, mPFC: medial prefrontal cortex.

According to the developers’ recommendations, the M values should not exceed 1.5 and all ncRNAs in all tissues analyzed with both common threshold and LinRegPCR settings met this requirement. Initial M values (with all ncRNAs included in the analysis), as computed by geNorm are reported in Supplementary Table S9. After step-wise exclusion of the worst ncRNA and pairwise variation for the determination of the optimal number of ncRNAs for normalization, the average expression stability of the remaining candidates was determined (Figure 4). In DRG, sno135 was identified as the least stable ncRNA; whereas the sno202/sno420 pair was the most stable (Figure 4A). In the dhSC, sno234 showed the lowest and the sno251/sno429 pair the highest stability (Figure 4B). In the mPFC, sno135 was determined as the least stable ncRNA and the sno142/sno202 pair as the most stable (Figure 4C). Pairwise variation analysis showed that in all cases the V values were below 0.15, suggesting that the best pair of ncRNAs would already be sufficient for appropriate normalization (Figures 4D–F).

### NormFinder

**FIGURE 5:**
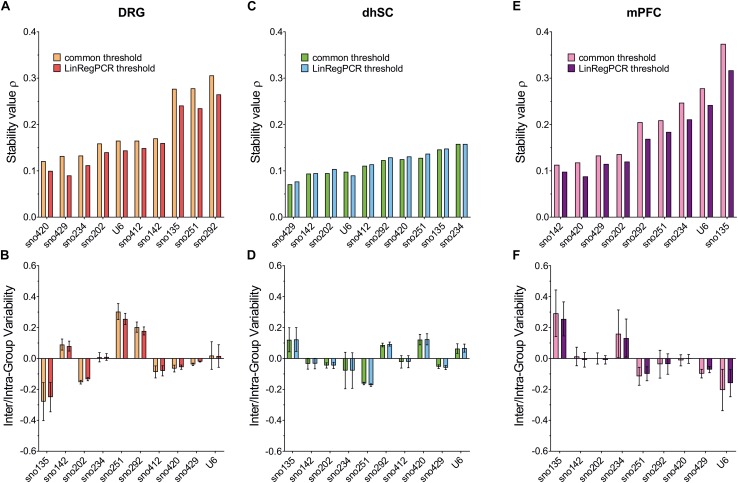
Stability value ρ (upper panel), inter- (box) and intra- (whiskers) group variability (lower panel), as evaluated by NormFinder for all evaluated ncRNAs in DRG **(A,B)**, dhSC **(C,D)**, and mPFC **(E,F)** of non-treated (*n* = 5), sham (*n* = 6), and SNI (*n* = 5) mice, as estimated by setting a common threshold (0.1) and by using LinRegPCR software. DRG: dorsal root ganglia, dhSC: dorsal horn of the spinal cord, mPFC: medial prefrontal cortex.

Stability values ρ, inter- and intra-group variability for all ncRNAs, were evaluated (Figure 5; detailed data are provided in Supplementary Table S10). Both threshold settings provided similar ranking results: In DRG, the lowest ρ value was observed for sno420 when using the common threshold (ρ = 0.121) and sno429 when using LinRegPCR (ρ = 0.090; Figures 5A,B). The highest ρ value was observed for sno292 (ρ = 0.306 for the common threshold and ρ = 0.265 with LinRegPCR). The sno234/sno429 pair was identified as the best pair combination (ρ = 0.069 and ρ = 0.048, respectively for the common threshold and LinRegPCR settings; Table 2). In the dhSC, the most stable ncRNA was sno429 (ρ = 0.071 using the common threshold and ρ = 0.077 using LinRegPCR; Figures 5C,D) and the best pair combination sno429/U6 (ρ = 0.062 for both settings; Table 2), whereas sno234 exhibited the worst performance (ρ = 0.158 for both settings). In the mPFC, the setting of a common threshold revealed that sno142 was the most stable ncRNA (ρ = 0.113), whereas sno420 showed the best performance when using LinRegPCR (ρ = 0.088; Figures 5E,F). The most stable pair was sno202/sno420 (ρ = 0.078) for the common threshold and sno142/sno420 (ρ = 0.070) for LinRegPCR (Table 2). Both the common threshold and LinRegPCR settings identified sno135 as the least stable ncRNA (ρ = 0.374 for the common threshold and ρ = 0.317 for LinRegPCR).

**TABLE 2:** The best combination of two ncRNAs and their stability values as computed by NormFinder in DRG, dhSC, and mPFC of non-treated, sham, and SNI mice, calculated by setting a common threshold (0.1) and by using LinRegPCR software.

	**DRG**		**dhSC**		**mPFC**	
			
	**0.1**	**LinReg**	**0.1**	**LinReg**	**0.1**	**LinReg**
Best combination of two genes	sno234/sno429	sno234/sno429	sno429/U6	sno429/U6	sno202/sno420	sno142/sno420
Stability value for best pair	0.069	0.048	0.062	0.062	0.078	0.070

*DRG, dorsal root ganglia; dhSC, dorsal horn of the spinal cord; mPFC, medial prefrontal cortex; SNI, spared nerve injury.*

### Overall Ranking of Potential Reference Genes for the Different Neuronal Tissues

**FIGURE 6:**
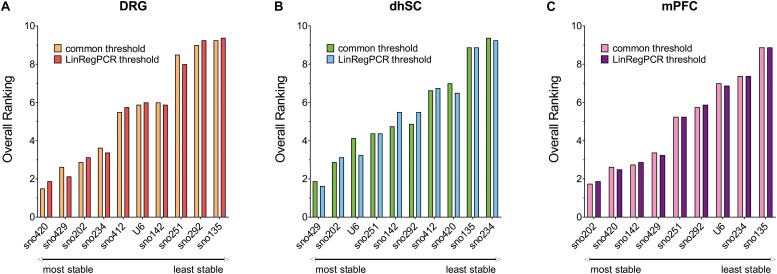
Overall ranking of all evaluated ncRNAs, as obtained by calculating the mean of the separate rankings achieved in BestKeeper, comparative delta-Cq method, geNorm, and NormFinder, in DRG **(A)**, dhSC **(B)**, and mPFC **(C)** of non-treated (*n* = 5), sham (*n* = 6), and SNI (*n* = 5) mice, as estimated by setting a common threshold (0.1) and by using LinRegPCR software. DRG: dorsal root ganglia, dhSC: dorsal horn of the spinal cord, mPFC: medial prefrontal cortex.

Each potential reference gene was assigned a rank, according to its performance (1 was assigned to the most stable ncRNA) in each analysis. For BestKeeper analysis, ncRNAs were ranked based on both their SD and correlation with the BKI. Finally, we calculated the arithmetic mean and compiled an overall ranking list (Figure 6 and Table 3). Since all four methods employed for assessing the stability of the ncRNAs, and their potential as reference genes, were taken into account as of equal importance, we did not use the geometric mean as it indicates the tendency of a set of values and dampens the effect of extreme values. Nevertheless, the rating was also calculated using the geometric mean and only sno251 in the dhSC for the common threshold exhibited a slightly different ranking (one rank higher), compared to the ranking obtained by using the arithmetic mean (Supplementary Table S11). Both the common threshold and LinRegPCR settings revealed the same three ncRNAs as the most stable and the same two candidates as the least stable, which, however, were different between the three neuronal tissues. Specifically, in DRGs, sno420, sno429, and sno202 ranked as the top three stable ncRNAs, whereas the lowest stability was observed for sno135, sno292, and sno251 (Figure 6A). In the dhSC, sno429, sno202, and U6 had the best stability, whereas sno234 and sno135 were the most variably expressed ncRNAs (Figure 6B). In the mPFC, the best three candidates were sno202, sno420, and sno142, whereas the least stable ncRNAs were sno135, sno234, and U6 (Figure 6C). The overall ranking for DRG and mPFC resembled the ranking provided by geNorm analysis. Furthermore, the overall ranking did not substantially differ between the common threshold and LinRegPCR datasets.

**TABLE 3:** Overall ranking of all evaluated ncRNAs expressed as the arithmetic mean of the ranks achieved in Bestkeeper, delta-Cq method, geNorm, and NormFinder in DRG, dhSC, and mPFC of non-treated, sham, and SNI mice, calculated by setting a common threshold (0.1) and by using LinRegPCR software.

**DRG**				**dhSC**					**mPFC**	
							
**ncRNA**	**0.1**	**LinReg**	**ncRNA**	**ncRNA**	**0.1**	**LinReg**	**ncRNA**	**ncRNA**	**0.1**	**LinReg**
							
	**Mean score**				**Mean score**				**Mean score**	
**sno420**	1.50	1.88	**sno420**	**sno429**	1.88	1.63	**sno429**	**sno202**	1.75	1.88
**sno429**	2.63	2.13	**sno429**	**sno202**	2.88	3.13	**U6**	**sno420**	2.63	2.50
**sno202**	2.88	3.13	**sno202**	**U6**	4.13	3.25	**sno202**	**sno142**	2.75	2.88
sno234	3.63	3.38	sno234	sno251	4.38	4.38	sno251	sno429	3.38	3.25
sno412	5.50	5.75	sno412	sno142	4.75	5.50	sno292	sno251	5.25	5.25
U6	5.88	5.88	sno142	sno292	4.88	5.50	sno142	sno292	5.75	5.88
sno142	6.00	6.00	U6	sno412	6.63	6.50	sno420	U6	7.00	6.88
sno251	8.50	8.00	sno251	sno420	7.00	6.75	sno412	sno234	7.38	7.38
sno292	9.00	9.25	sno292	sno135	8.88	8.88	sno135	sno135	8.88	8.88
sno135	9.25	9.38	sno135	sno234	9.38	9.25	sno234			

*DRG, dorsal root ganglia; dhSC, dorsal horn of the spinal cord; mPFC, medial prefrontal cortex; SNI, spared nerve injury; the three most stable ncRNAs are depicted in bold.*

### Reference Gene Selection Affects Outcome of miR-21a-5p Quantification

**FIGURE 7:**
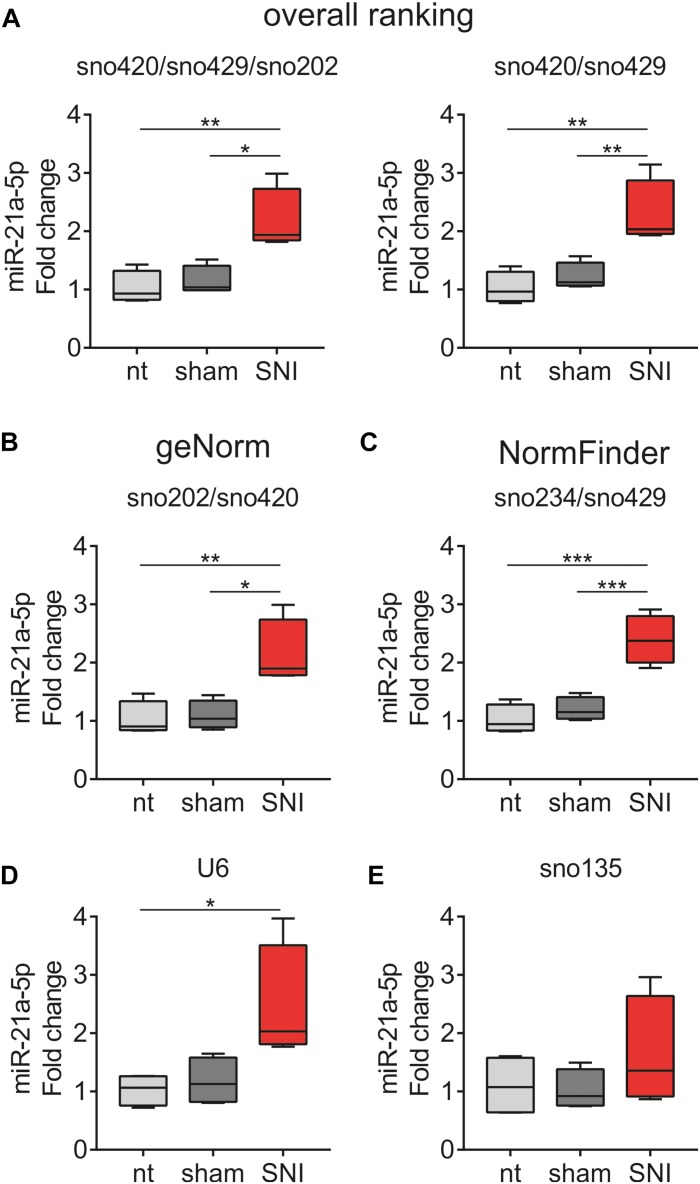
miR-21a-5p quantification in the DRG of non-treated (nt), sham and SNI mice using **(A)** three (sno420/sno429/sno202, left panel) or two (sno420/sno429, right panel) most stable ncRNAs from the overall ranking list, the most stable pair of ncRNAs as computed by **(B)** geNorm (sno202/sno420) and **(C)** NormFinder (sno234/sno429), **(D)** U6, and **(E)** sno135. miR-21a-5p levels were expressed as fold changes relative to the respective expression levels in the non-treated group using the 2^– ΔΔCq^ method. Values are presented as boxplots, representing upper quartile, median, and lower quartile, whereas whiskers depict minimum and maximum values.^∗^*p* < 0.05, ^∗∗^*p* < 0.01, ^∗∗∗^*p* < 0.001.

The ranked ncRNAs were assessed for their suitability to quantify miR-21a-5p, which is upregulated in DRG after peripheral nerve injury (Strickland et al., 2011; Zhou et al., 2015; Hori et al., 2016; Chang et al., 2017; Karl et al., 2017; Simeoli et al., 2017; Zhang Z. J. et al., 2018). miR-21a-5p was found upregulated in the SNI group when three (sno420/sno429/sno202; *F*_2_,_9_ = 10.72, *p* = 0.0042; non-treated vs. SNI *p* = 0.0058, sham vs. SNI *p* = 0.0110; Figure 7A, left panel) or two (sno420/sno429; *F*_2_,_9_ = 12.05, *p* = 0.0028; non-treated vs. SNI *p* = 0.0035, sham vs. SNI *p* = 0.0097; Figure 7A, right panel) most stable ncRNAs from the overall ranking list were used as reference genes. The most stable pair of genes as computed by geNorm (sno202/sno420; *F*_2_,_9_ = 9.781, *p* = 0.0055; non-treated vs. SNI *p* = 0.0086, sham vs. SNI *p* = 0.0121; Figure 7B) and NormFinder (sno234/sno429; *F*_2_,_9_ = 24.34, *p* = 0.0002; non-treated vs. SNI *p* = 0.0003, sham vs. SNI *p* = 0.0009; Figure 7C) provided similar results. However, when U6 was used as a normalizer, miR-21a-5p expression was different only between non-treated vs. SNI (*F*_2_,_9_ = 5.72, *p* = 0.0249; non-treated vs. SNI *p* = 0.0321; Figure 7D), whereas the use of sno135 completely abolished the significance (*F*_2_,_9_ = 1.048, *p* = 0.3897; Figure 7E). The use of single reference genes revealed that the five most stable ncRNAs, namely, sno420 (*F*_2_,_9_ = 11.06, *p* = 0.0038; non-treated vs. SNI *p* = 0.0052, sham vs. SNI *p* = 0.0104; Supplementary Figure S1A), sno429 (*F*_2_,_9_ = 11.21, *p* = 0.0036; non-treated vs. SNI *p* = 0.0039, sham vs. SNI *p* = 0.0153; Supplementary Figure S1B), sno202 (*F*_2_,_9_ = 8.319, *p* = 0.0090; non-treated vs. SNI *p* = 0.0160, sham vs. SNI *p* = 0.0158; Supplementary Figure S1C), sno234 (*F*_2_,_9_ = 33.09, *p* < 0.0001; non-treated vs. SNI *p* = 0.0001, sham vs. SNI *p* = 0.0002; Supplementary Figure S1D), and sno412 (*F*_2_,_9_ = 17.07, *p* = 0.0009; non-treated vs. SNI *p* = 0.0023, sham vs. SNI *p* = 0.0014; Supplementary Figure S1E) could also detect the upregulation of miR-21a-5p in the SNI mice in comparison to non-treated and sham mice. Normalization to sno142 showed a significant difference only between non-treated vs. SNI mice (*F*_2_,_9_ = 8.652, *p* = 0.0080; non-treated vs. SNI *p* = 0.0071; Supplementary Figure S1F), whereas sno251 (*F*_2_,_9_ = 16.63, *p* = 0.0010; non-treated vs. SNI *p* = 0.0010, sham vs. SNI *p* = 0.0058; Supplementary Figure S1G) and sno292 (*F*_2_,_9_ = 48.52, *p* < 0.0001; non-treated vs. SNI and sham vs. SNI *p* < 0.0001; Supplementary Figure S1H) exaggerated the upregulation of miR-21a-5p, indicating that these two ncRNAs likely were regulated by the treatment.

## Discussion

This study for the first time identified suitable reference genes for the normalization of miRNA expression in the SNI mouse model of peripheral nerve injury by employing four commonly used statistical tools, BestKeeper (Pfaffl et al., 2004), the comparative delta-Cq method (Silver et al., 2006), geNorm (Vandesompele et al., 2002), and NormFinder (Andersen et al., 2004), and two Cq datasets (one with a common threshold set at 0.1, in which qPCR efficiency for all amplicons was assumed to be 2 and the second one with LinRegPCR corrected baselines and efficiencies). We compiled a mean score ranking list, evaluating the expression stability of eleven ncRNAs regularly used for miRNA normalization in DRG, dhSC, and mPFC of naive, sham, and SNI mice. In DRG, the most stable ncRNAs were sno420, sno429, and sno202. In the dhSC, the best ncRNAs were sno429, sno202, and U6, whereas in the mPFC, the three superior ncRNAs were sno202, sno420, and sno142. sno55 was found unsuitable for further analysis due to its high variability and sno135 consistently ranked in the two least stable candidates. Overall, there were minor differences between the two Cq datasets and the final ranking indicated a general agreement on the most and least stable ncRNAs with minimal differences.

The efficiency of a qPCR reaction is regularly considered to be 2 (Bustin et al., 2013; Bustin and Nolan, 2017), particularly when commercially available hydrolysis probes are used. Through experimental examples, at least for mRNA expression, application manuals suggest that measuring efficiency is not required when using, e.g., Taqman^®^ gene expression assays, since they should perform at ∼100% ± 10% (Applied Biosystems, 2004, 2015). The gold standard for measuring the efficiency of assays and primer sets is the standard curve approach, in which Cq values are determined for reactions containing serial dilutions of the target template (Hellemans and Vandesompele, 2011). Although we did not run a standard curve in our experiments, we addressed the potential issue of variable efficiencies by analyzing individual amplifications with the LinRegPCR software (Ramakers et al., 2003; Ruijter et al., 2009, 2015; Tuomi et al., 2010) and compared the results with the results obtained by setting the threshold manually at 0.1, under the assumption that the efficiency was 2. Efficiencies obtained from LinRegPCR ranged between 1.765 and 1.888 and were slightly below the expected values. However, correlation analyses of efficiencies and initial template integrity as well as comparison of efficiencies vs different tissues and vs experimental groups were not statistically significant. Additionally, the overall ranking of all the candidates was similar when the efficiency was set to 2 vs. using the efficiencies calculated by LinRegPCR. The MIQE guidelines do not provide recommendations for appropriate PCR efficiency values and we cannot exclude that the lower efficiencies obtained from LinRegPCR could be attributed to the strict parameters set by the program or the presence of inhibitors in the reactions, even though all protocols were strictly implemented (Bustin et al., 2009). Nevertheless, the efficiencies did not correlate with RNA integrity or differ between experimental days and experimental groups, indicating that the obtained data were highly reliable.

Although there are other methods, such as miRNA sequencing and microarray analyses, RT-qPCR remains the most commonly used method to quantify miRNA expression due to its high sensitivity and specificity (Bustin and Nolan, 2017). However, appropriate normalization strategies are critically important for acquiring reliable results and ensuring reproducibility (Bustin et al., 2009; Bustin and Nolan, 2017). A suggested approach for normalization relies on multiple validated reference genes (Vandesompele et al., 2002; D’Haene et al., 2012). It is proposed that before initiating an RT-qPCR experiment, the most appropriate reference genes need to be determined for a given experimental setup using a representative set of samples (Hellemans and Vandesompele, 2011). However, RT-qPCR data are frequently normalized with only one reference gene (Bustin and Nolan, 2017). Despite their frequent use, the stability of these reference genes is not always documented for the particular experimental conditions and they may have even been reported to be unsuitable normalizers in specific experimental setups, e.g., U6 for miRNA expression in neuronal differentiation (Lim et al., 2011; Schwarzenbach et al., 2015). miRNAs are currently being explored as potential biomarkers for various diseases as well as tools for therapeutic interventions (Hayes et al., 2014; Ramanathan and Ajit, 2016; Lopez-Gonzalez et al., 2017; Zhou et al., 2018; Martinez and Peplow, 2019). Therefore, the establishment of appropriate reference genes for reliable and reproducible quantification of miRNA expression levels is of critical importance.

Neuropathic pain resulting from nerve injury is a serious health problem, which greatly decreases patients’ life quality and inflicts a high economic burden on society (Pain Proposal Steering Committee, 2010). It is considered to be one of the least manageable pain syndromes for which available treatment and medication is suboptimal (Van Hecke et al., 2014). Furthermore, due to the complex mechanisms involved in the development and maintenance of neuropathic pain, the use of animal models for elucidating the pain pathways is apparent (Jaggi et al., 2011; Gregory et al., 2013). Nerve injury and consecutive neurodegeneration or other complex pathophysiological reactions of the nervous system have been associated with miRNA deregulation both in humans and animal models (Bai et al., 2007; Kress et al., 2013; McDonald et al., 2014; Gong et al., 2015; Zhou et al., 2017) and several studies have addressed the suitability of deregulated miRNAs as potential biomarkers or targets for neurodegeneration and neuropathic treatments (Jiangpan et al., 2016; Ramanathan and Ajit, 2016; Lopez-Gonzalez et al., 2017; Birklein et al., 2018). However, the validity of studies quantifying miRNA expression in rodent pain models may be hampered by the use of suboptimal reference controls for miRNA normalization.

In the present study, we systematically evaluated the suitability of eleven ncRNAs for miRNA normalization in the SNI mouse model for peripheral nerve injury and neuropathy. Coding mRNA genes were excluded from our analysis as their much larger size adds variability in RNA extraction, reverse transcription, and qPCR efficiency. We assessed the stability of snoRNA and snRNA. Although these two classes of small RNAs have been extensively used for normalizing miRNAs, they differ from miRNAs in size, transcription, processing, and expression patterns (Meyer et al., 2010; Chugh and Dittmer, 2012). Moreover, both snRNAs and snoRNAs have been found deregulated in various human pathologies, such as different types of cancers, liver, heart and cardiovascular diseases, and disorders of the central nervous system (Madadi et al., 2019; Watson et al., 2019). The causes for differential expression of snRNAs and snoRNAs in health and disease are poorly understood (Didychuk et al., 2018; Kufel and Grzechnik, 2019). In humans, deletions in SNORD116C/D box (Duker et al., 2010) and HBII-85C/D box (Sahoo et al., 2008) are associated with Prader–Willi syndrome, whereas duplications within the same chromosome region (15q11-q13) are related to autism spectrum disorder (Hogart et al., 2009; Nakatani et al., 2009). Mutations in the gene encoding for RNA polymerase III deregulate snRNA and snoRNA expression (Azmanov et al., 2016). Adding to this complexity, snoRNAs have functions beyond ribosomal modification, e.g., they can act like miRNAs (Ender et al., 2008; Patterson et al., 2017) or as indirect transcriptional inhibitors by modifying RNA-binding proteins (Wang et al., 2008). Therefore, the use of these ncRNAs as reference genes for miRNA quantification is recommended only after validation of their stability in the specific experimental settings. The alternative to snoRNAs and snRNAs for the normalization of miRNAs is the global mean normalization or the use of miRNAs that mimic the global mean expression (Mestdagh et al., 2009; D’Haene et al., 2012). However, both of these approaches rely on the analysis of very large numbers of miRNAs, resulting in disproportionally high costs, particularly for small-scale studies.

The most commonly used ncRNA for normalizing miRNA expression levels in rodent nerve injury models is the snRNA U6 (Chen et al., 2018; Norcini et al., 2018; Pan et al., 2018; Yan et al., 2018; Jiang et al., 2019). Other ncRNAs that have been used as reference genes are the snoRNAs sno202 (Imai et al., 2011; Kusuda et al., 2011; Karl et al., 2017), sno135 (Norcini et al., 2018), and sno55 (Willemen et al., 2012). In most of the studies, the aforementioned controls are used as single normalizers. In our analysis, U6 was found in the top three stable ncRNAs only in the dhSC, whereas it ranked in the medium-to-low range in DRG and mPFC. In DRG, although U6 showed the smallest SD among the samples, it scored at the low end when it was compared to the BKI and average or below average in the delta-Cq method, geNorm, and NormFinder, thus representing an ncRNA of medium–low expression stability. In the mPFC, the rank of U6 was similar to DRG. Overall, among all candidates investigated in the present study, U6 consistently ranked slightly below average, suggesting that it may not be the most suitable candidate for miRNA normalization, in particular when used as a single normalizer. Interestingly, U6 has already been documented to be unsuitable for normalizing miRNAs in serum (Benz et al., 2013), plasma (Tang et al., 2015; Gevaert et al., 2018), different types of cancers (Lou et al., 2015; Popov et al., 2015), and in neuronal differentiation (Lim et al., 2011; Schwarzenbach et al., 2015).

sno55 (Snord110) and sno135 (Snord65) showed considerable variability and this confirmed previous reports suggesting that both snoRNAs do not represent stable reference genes for miRNA normalization (Karl et al., 2017). In contrast, sno202 (Snord68) and sno234 (Snord70) have been reported to be stably expressed across 12 different naive mouse tissues (Applied Biosystems, 2010). In line with these findings, the overall ranking for sno202 in our analysis was in the top 30% for all three tissues assessed, suggesting that sno202 is one of the most suitable candidates for normalizing miRNA expression. Additionally, sno202 was the only candidate that ranked in the three most stable ncRNAs in all tissues assessed. sno202 has been stated to be stable in the chronic inflammatory (Kusuda et al., 2011), axotomy (Kusuda et al., 2011), acute noxious stimulation (Kusuda et al., 2011), and SNI pain models (Kusuda et al., 2011; Karl et al., 2017), whereas it is highly unstable in the livers of mice subjected to an obesity model (Matouskova et al., 2014). On the other hand, in our analysis, sno234 ranked fourth in DRG, last in dhSC, and second to last in the mPFC, although it was documented as one of the most stable ncRNAs in the aforementioned study on obesity (Matouskova et al., 2014).

The differences in the stability of the evaluated ncRNAs were further demonstrated by normalizing miR-21a-5p, a miRNA that is upregulated in the DRG after peripheral nerve injury, including spinal nerve ligation (Sakai and Suzuki, 2013; Chang et al., 2017; Zhang Z. J. et al., 2018), sciatic nerve transection (Zhou et al., 2015), partial sciatic nerve ligation (Hori et al., 2016), sciatic nerve dissection (Strickland et al., 2011), and SNI (Karl et al., 2017; Simeoli et al., 2017). The ncRNAs possibly suitable as single normalizers, demonstrating significant deregulation of miR-21a-5p in the DRG of SNI mice belonged to the five most stable ncRNAs (sno420, sno429, sno202, sno234, and sno412). ncRNAs with lower ranking either detected a significant regulation only when the non-treated vs. SNI groups were compared (U6 and sno142) or exaggerated the effect (sno251 and sno292), indicating that the latter ncRNAs are regulated by SNI; or in the case of sno135 failed to reveal any significant upregulation. These findings further support guidelines discouraging the use of single reference genes (Vandesompele et al., 2002; Bustin et al., 2009; D’Haene et al., 2012). In addition, the four methods indicate relative stability since they rely on the comparison between the analyzed ncRNAs. In order to address this issue, we quantified miR-21a-5p by using as reference genes three or two very stable ncRNAs as well as the pairs of ncRNAs proposed as most stable by geNorm and Normfinder, which provided similar results. Therefore, the use of at least two of the top four-to-five most stably expressed ncRNAs significantly improves reliability of miRNA quantification in the DRG. Our results, alongside the literature, provide further support that the stability of reference genes may dramatically vary between different tissues and different experimental conditions. The ncRNAs we have identified as stable should be considered stable in the assessed tissues, under the specific conditions used for RNA extraction, cDNA preparation and qPCR reactions, and the employed animal model. Thus, in other animal models for nerve lesion or under different sample handling procedures, these ncRNAs may not be the most appropriate for miRNA normalization and their stability should be re-evaluated. Nevertheless, our results provide a firm indication on which of these ncRNAs could be stably expressed and pilot experiments to select the most stable ncRNAs for specific experimental settings are highly recommended.

We for the first time identify highly stable reference genes suitable for miRNA quantification in the DRG, dhSC, and mPFC of non-treated, sham, and SNI mice. Our results underline the need for thorough validation of reference genes for miRNA normalization in different tissues and experimental settings. We expect that the ncRNAs reported here could be suitable for miRNA quantification in nervous tissues in other nerve lesion models, thus promoting validity and reproducibility in miRNA expression analyses.

## Data Availability Statement

All datasets generated for this study are included in the article/Supplementary Material.

## Ethics Statement

The animal study was reviewed and approved by the Austrian National Animal Experiment Ethics Committee of the Austrian Bundesministerium für Wissenschaft und Forschung (permit number BMWF-66.011/0054-WF/V/3b/2015).

## Author Contributions

TK, KK, and MK designed the study. TK, KK, and MM performed the sample collection and experiments. TK and KK performed the data analysis and interpreted the data. TK and MK wrote the manuscript. KK and MM critically reviewed the contents of the manuscript and suggested substantial improvements. All authors have approved the final version of the manuscript.

## Conflict of Interest

The authors declare that the research was conducted in the absence of any commercial or financial relationships that could be construed as a potential conflict of interest.

## Supplementary Material

FIGURE S1 miR-21a-5p quantification in the DRG of non-treated (nt), sham, and SNI mice using each assessed ncRNA as a single reference gene **(A)** sno420, **(B)** sno429, **(C)** sno202, **(D)** sno234, **(E)** sno412, **(F)** sno142, **(G)** sno251, and **(H)** sno292. miR-21a-5p levels were expressed as fold changes relative to the respective expression levels in the non-treated group using the 2^–ΔΔCq^ method. Values are presented as boxplots, representing upper quartile, median, and lower quartile, whereas whiskers depict minimum and maximum values. ^∗^*p* < 0.05, ^∗∗^*p* < 0.01, ^∗∗∗^*p* < 0.001, ^∗∗∗∗^*p* < 0.0001.

TABLE S1 MIQE checklist for authors, reviewers, and editors.

TABLE S2 Quantity and integrity of RNA extracted from DRG, dhSC, and mPFC of non-treated, sham, and SNI mice.

TABLE S3 Mean qPCR efficiencies for all amplified ncRNAs calculated by LinRegPCR.

TABLE S4 Spearman’s correlation between RNA integrity and qPCR efficiency per reaction.

TABLE S5 Kruskal–Wallis test for differences in efficiencies vs. experimental day **(A)** and vs. experimental groups **(B)**.

TABLE S6 Descriptive statistics of the Cq values for all evaluated ncRNAs in DRG **(A)**, dhSC **(B)**, and mPFC **(C)** of non-treated, sham, and SNI mice, calculated by setting a common threshold (0.1) and by using LinRegPCR software.

TABLE S7 Descriptive statistics of the Cq values and correlation analysis obtained from BestKeeper for all evaluated ncRNAs in DRG **(A)**, dhSC **(B)**, and mPFC **(C)** of non-treated, sham, and SNI mice, calculated by setting a common threshold (0.1) and by using LinRegPCR software.

TABLE S8 Comparison of all evaluated ncRNAs using the comparative delta-Cq method in DRG **(A)**, dhSC **(B)**, and mPFC **(C)** of non-treated, sham, and SNI mice, calculated by setting a common threshold (0.1) and by using LinRegPCR software.

TABLE S9 Initial M values of all evaluated ncRNAs as computed by geNorm in DRG, dhSC, and mPFC of non-treated, sham, and SNI mice, calculated by setting a common threshold (0.1) and by using LinRegPCR software.

TABLE S10 ρ values, inter- and intra-group variations for all evaluated ncRNAs as obtained from NormFinder in DRG **(A)**, dhSC **(B)**, and mPFC **(C)** of non-treated, sham, and SNI mice, calculated by setting a common threshold (0.1) and by using LinRegPCR software.

TABLE S11 Overall ranking of all evaluated ncRNAs expressed as the geometric mean of the ranks achieved in Bestkeeper, delta-Cq method, geNorm, and NormFinder in DRG, dhSC, and mPFC of non-treated, sham, and SNI mice, calculated by setting a common threshold (0.1) and by using LinRegPCR software.
